# How PM_2.5_ drives dementia: emerging mechanisms and unanswered questions

**DOI:** 10.1093/lifemedi/lnag007

**Published:** 2026-02-02

**Authors:** Alexia D Defrancesco, Chenju Yi, Brian G Oliver, Hui Chen

**Affiliations:** School of Life Sciences, Faculty of Science, University of Technology Sydney, Ultimo, NSW 2007, Australia; Department of Geriatrics, The Seventh Affiliated Hospital of Sun Yat-sen University, Shenzhen 518107, China; Guangdong Provincial Key Laboratory of Digestive Cancer Research, Shenzhen 518107, China; Shenzhen Key Laboratory of Chinese Medicine Active Substance Screening and Translational Research, Shenzhen 518107, China; School of Life Sciences, Faculty of Science, University of Technology Sydney, Ultimo, NSW 2007, Australia; Woolcock Institute of Medical Research, University of Macquarie, Macquarie Park, NSW 2113, Australia; School of Life Sciences, Faculty of Science, University of Technology Sydney, Ultimo, NSW 2007, Australia; Woolcock Institute of Medical Research, University of Macquarie, Macquarie Park, NSW 2113, Australia

Emerging evidence has highlighted the significant role that fine particulate matter (PM_2.5_) plays in the development of cognitive impairment and dementia, posing a growing public health challenge worldwide. This perspective explores the emerging and under-investigated mechanisms by which PM_2.5_ exposure contributes to neurodegenerative processes, such as the lung–brain axis and epigenetic regulation, highlighting the unmet need for targeted research and intervention strategies.

## The air we breathe, the minds we lose

Recent projections estimate 152.8 million people will be living with dementia by 2050 [1]. Air pollution, especially fine particulate matter (< 2.5 µm, PM_2.5_), is now recognized as a significant environmental risk for cognitive decline and dementia. The WHO has warned that 99% of the global population breathes polluted air exceeding the WHO guideline limits. While the cardiovascular and respiratory dangers of PM_2.5_ are well known, its insidious effects on the brain, across all ages, are only beginning to be understood.

PM_2.5_ are small enough to penetrate deep into the lungs and cross into the bloodstream, reaching organs including the brain. Sources are both outdoor (traffic, industry, agriculture, wildfires) and indoor (cooking, heating, smoking), with indoor exposure levels often exceeding outdoor levels in developing regions. The neurotoxicity of PM_2.5_ depends greatly on its source and/or chemical compositions, with heavy metals (e.g. lead and arsenic) and organic compounds (e.g. polycyclic aromatic hydrocarbons) posing greater neurotoxic risk. Notably, PM_2.5_ from traffic and biomass burning are most strongly linked to dementia risk [2]. PM_2.5_ can form toxic secondary particles through chemical reactions with other air pollutants, such as NO_2_ and ozone. For example, NO_2_ can react with ammonia, a major contributor to PM_2.5_ in agricultural emissions, to form ammonium nitrate particles. Large cohort studies across North America, Europe, and Asia consistently show that even small increases in ambient PM_2.5_ levels are associated with higher rates of dementia and Alzheimer’s disease (AD). The risk is not limited to the elderly: children exposed to high levels of PM_2.5_ also show impaired memory and attention, and young-onset dementia is rising [2]. Importantly, improvements in air quality are linked to reduced dementia risk, highlighting the potential for prevention.

## Classical mechanisms: neuroinflammation and oxidative stress

**Figure 1.**
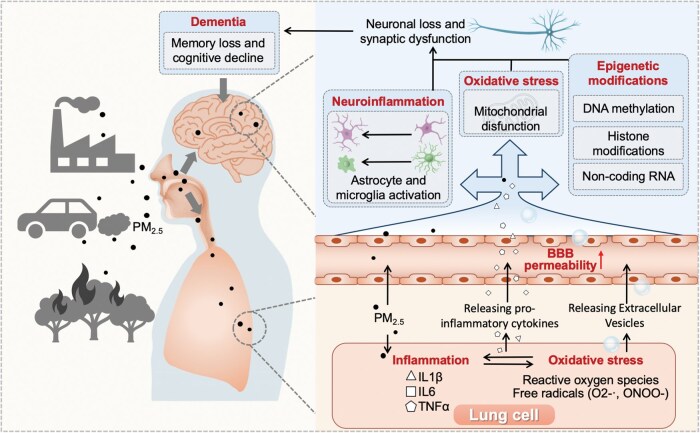
Proposed mechanisms of how PM_2.5_ inhalation causes the development of dementia.

PM_2.5_ can reach the brain via two main routes: a small amount through the nose via the olfactory and trigeminal nerve, or via the systemic circulation following absorption in the lungs (Fig. 1). In both scenarios, PM_2.5_ activate the cells in the respiratory tract (nose or lungs), resulting in systemic inflammation. The relative contribution of PM_2.5_ in the brain versus systemic inflammation in inducing neuroinflammation is unknown, but the reality is that both occur simultaneously and are likely to have additive/synergistic effects. In the brain, most research has focused on how PM_2.5_ damage the blood–brain barrier, activates microglia and astrocytes, resulting in oxidative stress and chronic inflammation (Fig. 1). This microenvironment accelerates the accumulation of amyloid-beta (Aβ) plaques and hyperphosphorylated tau, neuronal loss and synaptic dysfunction, key features of AD [2]. Mitochondrial dysfunction and impaired antioxidant defences further exacerbate injuries [2].

## Emerging mechanisms: the new frontiers

### The lung–brain axis

A paradigm shift is underway: the lung is not just a passive entry point for PM_2.5_, but an active player in brain health. Inhaled PM_2.5_ causes ongoing inflammatory response and oxidative stress in the airways and alveoli, releasing cytokines (such as IL-6, TNFα, and IL-1β) and other mediators into the bloodstream that can cross the blood–brain barrier and activate microglia and astrocytes to cause neuroinflammatory responses (Fig. 1). This ‘lung–brain axis’ is supported by evidence linking chronic lung diseases, such as chronic obstructive pulmonary disease (COPD) and cognitive decline [3]. Indeed, patients with COPD have an increased prevalence of stroke, white matter lesions, and memory decline. As such, the lung is a key gateway in the neurological impacts of air pollution, drawing attention to the intricate, multi-organ pathways through which inhaled toxins can progressively influence brain function. Key mechanisms linking the lung–brain axis to PM2.5 toxicity remain unclear, and further study could identify ways to prevent pulmonary and neurological damage.

### Extracellular vesicles (EVs): messengers of damage

EVs, tiny, lipid-bound particles released by cells, are emerging as key mediators of PM_2.5_ toxicity. EVs serve as mediators of intercellular communication and can influence the metabolism and function of recipient cells, with roles in a variety of diseases. In the brain, EVs can help remove toxic proteins and aggregates between cells, but they can also transfer these harmful materials to healthy cells, thereby assisting in both cellular detoxification and the spread of disease pathology, such as the intercellular delivery of misfolded tau and Aβ proteins linked to AD. Although the structure of the blood–brain barrier aims to limit the transportation of molecules via either transcellular or paracellular pathways, it has been proposed that brain-derived EVs carrying Aβ or/and phosphorylated-tau may still cross over to the circulation, as plausible biomarkers for early diagnosis. On the other hand, harmful EVs in the blood may also enter the brain via similar mechanisms. For example, in the lung, EVs are produced as part of an inflammatory response to air pollution, which has been proposed to drive smooth muscle dysfunction in asthma [4]. Upon exposure to PM_2.5_, lung cells release EVs loaded with bioactive cargo, including microRNAs (miRNAs), proteins, lipids, and even injured mitochondria and mitochondrial DNAs. These EVs are also likely to travel through the bloodstream, cross the blood–brain barrier, and deliver their cargo to brain cells, potentially spreading inflammation, oxidative stress and promoting neuronal injury and neurodegeneration (Fig. 1). As such, EVs and their cargo may offer new avenues for novel diagnostic panels and therapeutic strategies. However, how EVs cross the blood–brain barrier from either the brain or blood side remains a significant knowledge gap, which warrants further research.

### Epigenetic regulation: lasting imprints

Long-term PM_2.5_ exposure can alter gene expression in the brain through epigenetic modifications (DNA methylation, histone modifications, and miRNA regulation) that turn on/off genes without affecting the DNA sequence. For example, PM_2.5_ can induce hypomethylation of genes involved in Aβ production or alter histone acetylation linked to early AD pathology [5]. Such modifications also appear in individuals with amnestic mild cognitive impairment, suggesting its potential role as an early biomarker of AD. Other markers consistently displayed in humans and animals exposed to PM_2.5_ include hypermethylation of *SORBS2*, *BDNF*, and *SHANK3* genes, reduced histone methylation (e.g. H3K9me2 and H3K9me3), and altered miRNA (e.g. miR-21a and miR-222). Sex-specific effects have also been observed, with female mice showing resilience to prenatal PM_2.5_ exposure, which may be due to upregulated X-chromosome-linked histone demethylases Kdm5c and Kdm6a [2]. However, it is unclear whether it is because females have two copies of the X chromosome or the Y chromosome equivalent fails to respond to in-utero PM_2.5_ exposure, which requires further investigation.

Certain miRNAs are highly expressed in the brain and play essential roles in neural development, synaptic activity, and neuroinflammation. However, excess brain levels of miR-146a, playing a role in immune responses, have been linked to the *APOE ε4* gene variant-related risk of cognitive impairment, a significant genetic risk factor for AD. Disruption of the normal patterns of these miRNAs is increasingly recognized as a downstream effect of PM_2.5_ exposure on the induction of neurodegenerative diseases. In animal models, PM_2.5_ exposure can activate NF-κB signalling, leading to increased miR-574-5p, which impairs synaptic function and cognition. Similarly, other inflammation-related miRNAs may also promote neuroinflammatory pathways triggered by PM_2.5_. In addition, miRNAs transported within EVs released from the lungs may facilitate communication between the lung and brain cells, as a driver of the lung–brain axis regulation of PM_2.5_-induced neurotoxicity and cognitive impairment, thereby promoting the development and progression of AD-related pathology. For example, the above-mentioned miR-21a and miR-222 have been proposed as lung–brain axis miRNAs. PM_2.5_ exposure-induced lung EVs also contain let-7i-5p [4], which can inhibit genes (e.g. *Frizzled* 3, *GluN3A*) that are key for neural development and synaptic function.

## Future directions: unanswered questions

Despite growing recognition of the lung–brain axis, research has primarily focused on cigarette smoking-induced COPD, with relatively little attention given to the mechanisms involved in air pollution-associated disease. One particularly promising direction lies in the role of EVs and their miRNA cargo driving the lung–brain axis in mediating communication between the lung pathologies and the adverse brain responses that impair cognitive functions of all ages. miRNAs, known for their capacity to carry regulatory signals across organ systems, may represent a key mechanism through which exposure to ambient PM_2.5_ translates into central nervous system dysfunction. This highlights an urgent need to further characterize how age, developmental stage, and genetic predisposition interact with environmental exposures to influence cognitive trajectories.

## Conclusion: a call to action

The evidence is clear: PM_2.5_ is a major, modifiable risk factor for dementia across the lifespan. While classical mechanisms like neuroinflammation and oxidative stress remain important, new research into the lung–brain axis driven by EVs and epigenetic regulation is transforming our understanding of how air pollution harms the brain. To foster the development of new diagnostic biomarkers or treatment strategies, the research priority needs to be on how EVs and miRNAs cross the blood–brain barrier from both the blood and brain sides, and how to trace them to their source cells. In addition, stem cell-derived EVs may be modified to deliver epigenetic regulators to the brain to either promote neurogenesis or reverse senescence. Urgent investment in these emerging areas is needed, not only to unravel the mysteries of dementia but also to develop targeted strategies that protect brain health in a polluted world.
